# Validated RP‐HPLC–UV Method for the Quantitative Analysis and Stability Evaluation of Amoxicillin in Paediatric Gummy Tablets

**DOI:** 10.1002/elps.70103

**Published:** 2026-05-03

**Authors:** Samuele Bonafè, Anna Imbriano, Cinzia Pagano, Anna Migni, Elisa Bianconi, Laura Mercolini, Luana Perioli, Roccaldo Sardella

**Affiliations:** ^1^ Department of Pharmaceutical Sciences University of Perugia Perugia Italy; ^2^ Department of Pharmacy and Biotechnology (FaBiT), Alma Mater Studiorum, University of Bologna Bologna Italy

**Keywords:** amoxicillin, gummy tablets, method validation, paediatric dosage form, reversed‐phase high‐performance liquid chromatography coupled with ultraviolet (RP‐HPLC–UV), stability study

## Abstract

The development of products that encounter compliance from paediatric patients represents one of the challenges of pharmaceutical technology that aims to develop formulations able to overcome problems related to palatability and dosing flexibility and, in general, to patient acceptance. In this study, a fully validated reversed‐phase high‐performance liquid chromatography coupled with ultraviolet detection (RP‐HPLC–UV) method was applied for the quantitative determination of amoxicillin (AMX) in an innovative gummy tablet formulation designed for paediatric use. The chromatographic method was optimized and validated **largely (but not strictly) in accordance** with ICH Q2(R1) guidelines, evaluating specificity, linearity, limit of quantification (LOQ), trueness, precision, robustness and ruggedness. A **blank** matrix derived from a **blank** formulation was employed to closely simulate the sample matrix during method validation and quantitative analysis. The method exhibited excellent linearity over the concentration range of 0.0039–0.25 mg/mL (*R* ^2^ = 0.99) with satisfactory trueness (recoveries between 101.3% and 102.4%) and high precision (coefficient of variation [CV] ≤ 1.6%). The LOQ was estimated between 0.0020 and 0.0039 mg/mL. Extraction recovery was **also** evaluated using penicillin G as a model compound due to its chemical and physicochemical similarity to AMX, yielding a recovery of 97.0% ± 0.1%. Application of the validated method to AMX‐loaded gummy tablets confirmed a drug content of 94.7% ± 3.7% of the theoretical value. Stability studies, conducted over 6 months under different storage conditions, revealed a progressive decrease in AMX content within the gummy matrix with more pronounced degradation observed under vacuum at 25°C compared to storage in a dry cabinet at room temperature (RT). Overall, the developed RP‐HPLC–UV method proved to be reliable and suitable for routine quality control and stability assessment of AMX‐containing gummy tablets, supporting further development of this child‐friendly dosage form.

## Graphical abstract

A validated RP‐HPLC–UV method was successfully applied for amoxicillin determination in gummy tablets. The method is useful for an accurate amoxicillin content determination in paediatric gummies and was applied to study the stability profile of the drug, evaluated over 6 months under storage conditions.

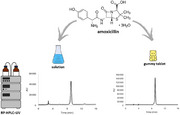

## 1 Introduction

The development of pharmaceutical formulations that encounter compliance from paediatric patients remains a significant challenge for the pharmaceutical industry. This issue is particularly pronounced in the context of antibiotic therapies, where patient adherence is frequently compromised by the limited acceptability of currently available dosage forms. Poor palatability, inconvenient administration and difficulties of correct dosing often result in reduced compliance, especially in paediatric populations [1].

Amoxicillin (AMX) is a broad‐spectrum β‐lactam antibiotic, extensively prescribed in paediatric practice for the treatment of a wide range of bacterial infections. Although AMX possesses an inherently bitter taste, the main AMX dosage forms currently available on the market are oral formulations as suspensions and tablets. These preparations are recommended in paediatrics for the excellent bioavailability, accompanied by simplicity and non‐invasiveness of administration, fundamental aspects in paediatric therapies. On the other hand, these oral formulations are often poorly accepted by children often due to their unpleasant taste [1]. In this context, gummy tablets could represent a promising alternative to conventional pharmaceutical dosage forms, as they have the potential to improve palatability, ease of administration and overall patient acceptance.

The development of age‐appropriate paediatric formulations requires careful consideration of several critical factors, including ease of administration, dose flexibility based on body weight, palatability and adequate stability under exposure to light, heat and humidity [2, 3, 4]. Recently, starch‐based AMX‐loaded gummy tablets were successfully developed using three‐dimensional (3D) printing technique, demonstrating the feasibility of producing innovative paediatric formulations with customizable dosing [5, 6]. In the perspective to purpose a valuable alternative to conventional formulations, it is very important to assess that once formulated in the gummy tablet, AMX maintains its original features over the time.

Thus, the development of robust and reliable analytical methods for the determination of AMX in different matrices is essential to guarantee the efficacy as well as the quality of the product. In addition to these applications, suitable analytical methodologies are also required for routine quality control of commercially available products, as well as for the characterization and evaluation of newly developed AMX‐containing formulations.

High‐performance liquid chromatography (HPLC) is by far the most widely employed analytical technique for the analysis of AMX [7]. Many studies reported in literature describe the development and validation of HPLC methods for the quantification of AMX in a variety of pharmaceutical formulations.

Most of these methods are performed under reversed‐phase (RP) chromatographic conditions, typically using octadecyl silica (C18) columns coupled with conventional spectrophotometric detectors [8, 9, 10], and are applied to the analysis of different dosage forms such as tablets, capsules and oral suspensions [11, 12]. Although less common, alternative HPLC approaches for AMX analysis have also been reported, including methods based on different separation mechanisms such as strong cation exchange chromatography [13]. Furthermore, variability exists in the choice of detection systems, with some studies employing detectors other than traditional ultraviolet (UV)–Vis spectrophotometry, such as mass spectrometry (MS), to enhance sensitivity and selectivity [14].

Going into detail, Unutkan et al. described a sensitive HPLC–UV method for the quantification of AMX in commercial pharmaceutical products and environmental matrices, obtaining excellent linearity (correlation coefficient of 0.9999), low limits of detection and successful application to solid and liquid oral dosage forms such as tablets and suspensions [11]. These findings further support the reliability and broad applicability of HPLC methods for routine pharmaceutical quality control. Other investigations have aimed at enhancing method selectivity and enabling stability studies. In this context, Konari and Jacob developed a stability‐indicating RP‐HPLC method for the simultaneous analysis of AMX and flucloxacillin in pharmaceutical dosage forms. Their method demonstrated high specificity toward degradation products formed under various stress conditions, including acidic, alkaline, oxidative and photolytic degradation [12]. This study underlines the relevance of stability‐indicating analytical methods able to effectively discriminate the active pharmaceutical ingredient (API) from degradation compounds and formulation excipients.

Recent advances in analytical method development have also emphasized the importance of environmentally sustainable chromatographic procedures and multi‐component analytical strategies. In this regard, Almalki et al. introduced a green HPLC‐MS method for the determination of AMX and its related substances, focusing on minimizing organic solvent consumption and improving the environmental profile of the analytical procedure [14].

Altogether, these studies highlight the ongoing progress in HPLC method development toward shorter analysis times, higher sensitivity and improved environmental sustainability. Nevertheless, despite the wide range of available analytical methods, there is still a need for the development of simple, rapid and robust HPLC procedures characterized by reduced analysis time, straightforward sample preparation and validation according to international agencies’ requirements. Furthermore, the variability in chromatographic parameters reported in the literature, such as mobile phase composition, detection wavelength and validation protocols, indicates the necessity for further optimization depending on the specific pharmaceutical formulation and intended analytical application.

In the present study, as part of a broader research project aimed at optimizing AMX formulations for paediatric use, the application of a fully validated HPLC–UV analytical method for the quantitative analysis of AMX in gummy tablet formulations is described. The stability of the produced gummy tablets was also investigated over a defined period under controlled conditions.

## 2 Materials and Methods

### 2.1 Chemicals and Reagents

HPLC‐grade acetonitrile (ACN), penicillin G, calcium chloride anhydrous and potassium phosphate dibasic trihydrate (K_2_HPO_4_·3H_2_O) were purchased from Merck Life Science (Merck KGaA, Darmstadt, Germany). Water for HPLC analysis was purified with a Milli‐Q Plus 185 system from Millipore (Milford, MA, USA). The mobile phase components were degassed by sonication for 10 min before use. Corn starch and AMX trihydrate were purchased from ACEF S.p.A. (Fiorenzuola d'Arda, Italia). Organic acacia honey was produced by hives on a local farm and was bought in a local market (Perugia, Italia).

The simulated gastric fluid (SGF) was prepared according to Farmacopea Ufficiale Italiana (FU XII Ed.), and the composition is as follows: 2 g NaCl, 80 mL HCl 1 M, and bidistilled water until 1000 mL.

### 2.2 Instrumentation and Chromatographic Conditions

HPLC analyses were performed on a Shimadzu (Kyoto, Japan) LC‐20A Prominence, equipped with a CBM‐20A communication bus module, two LC‐20 AD dual piston pumps, an SPD‐M20A photodiode array detector and a Rheodyne 7725i injector (Cotati, CA, USA) with a 20 µL stainless steel loop. An **Inertsil** ODS‐3 column (GL Sciences Inc., Japan) 250 × 4.6 mm^2^ i.d., 5 µm, 100 Å pore size was used. The employed column was conditioned at a 1.0 mL/min flow rate for at least 60 min before use, with the selected mobile phase. Column temperature was controlled through a Phenomenex (Torrance, CA, USA) Thermasphere HPLC column chiller/heater (Model TS‐430) thermostat. The injection volume was set at 20 µL, and the chromatographic analyses were followed at 230 nm. This wavelength of detection was selected according to the literature [8, 11]. A mixture of K_2_HPO_4_·3H_2_O 0.1 M in water/ACN (90:10, v/v; pH of the aqueous phase equal to 8.70) was identified as the best mobile phase for the purpose of the study.

The flow rate was set at 1.0 mL/min, whereas the column temperature was set at 25°C. After each analysis, the column was subjected to a blank injection to appraise an incidental carry‐over from the previous analysis. In order to evaluate the column performance, NaNO_2_ from Merck Life Science (Merck KGaA, Darmstadt, Germany) was used as the unretained marker. Sodium nitrite has been used to estimate dead time in the employed RP system due to its negligible retention. Indeed, it offers distinct advantages such as high aqueous solubility, low cost and UV detectability. However, detection at low wavelengths and potential minor secondary interactions may limit robustness compared to conventional markers. Nevertheless, despite these limitations, sodium nitrite may represent a suitable compromise as an unretained marker in RP chromatography applications employing C18 columns.

### 2.3 Gummy Tablets Preparation

The gummy tablets were prepared by casting method starting from a gel whose composition has been optimized in a previous work [5] and consists of acacia honey 30% w/w, corn starch 10% w/w and ultrapure water until 100% w/w. Honey was dissolved in ultrapure water under magnetic stirring (600 rpm) for 10 min at room temperature (RT). Then, corn starch was dispersed in the prepared solution. The obtained dispersion was stirred (400 rpm) and maintained in a water bath at 80°C in order to induce starch gelation. After gelation occurred, the gel was kept under stirring for 10 min. Following, the gel was left to cool at RT under magnetic stirring (100 rpm). Loaded gummy tablets were prepared by dispersing 2% w/w AMX powder in the prepared cooled gel by a Cito Unguator, for 2 min at 2000 rpm. Then the loaded gel was poured into ‘teddy bears’ silicon moulds and left to dry under CaCl_2_ at RT for 72 h. Subsequently, the gummy tablets were removed from the moulds and left under CaCl_2_ for further 24 h. After drying, they were stored individually under vacuum packages. Considering that for 50 g of gel, 34 gummy tablets were obtained, the final theoretical AMX loading was 29.4 mg/single‐dose unit. For the validation procedure, penicillin G‐loaded gummy tablets were prepared. The gel was prepared as described above. In order to know the exact amount of drug/unit, each gummy tablet was loaded as follows: A fixed amount of penicillin G was manually dispersed in a fixed amount of gel (1.47 g/single‐dose unit) using a spatula until homogeneity. Then, the gel was cast in the ‘teddy bear’ silicon mould and dried as described above.

### 2.4 Method Validation

The developed HPLC method was validated largely (but not strictly) in accordance with ICH Q2(R1) guidelines and based on a previous study by some of the present authors [15]. The following validation parameters were evaluated: specificity, linearity, limit of quantitation (LOQ), trueness, precision (repeatability and intermediate precision), robustness and ruggedness. In addition, an accuracy profile was established. Method validation was performed using a conventional UV–visible detector.

Method specificity was assessed by evaluating potential interferences from formulation excipients at the retention time of AMX trihydrate (approximately 9 min). For this purpose, a blank formulation (identical in composition to the drug product except for the absence of AMX) was analysed. The blank formulation was cut into smaller portions and maintained under magnetic stirring in 1 L of double‐distilled water for 24 h. Subsequently, an aliquot (approximately 15 mL) of the resulting suspension was centrifuged at 4000 rpm for 10 min, and the supernatant was collected. A 0.5 mL aliquot of the supernatant was diluted with an equal volume of mobile phase and filtered through a 0.45 µm nylon syringe filter to remove any insoluble particles prior to RP‐HPLC analysis, which was performed according to the procedure described in Section 2.2.

Quantitative determination of AMX in the pharmaceutical formulation was carried out using a seven‐point calibration curve. A standard stock solution of AMX (0.5 mg/mL) was prepared and serially diluted to obtain the following concentrations: 0.0039, 0.0078, 0.0156, 0.0313, 0.0625, 0.125 and 0.25 mg/mL. All calibration solutions were prepared in a 50:50 (v/v) mixture of mobile phase and extraction solution. To better simulate the actual sample matrix, the extraction solution obtained from the blank formulation (hereafter referred to as the blank matrix) was incorporated into all preparations. A target test concentration of 0.05 mg/mL was selected. Prior to analysis, all solutions were sonicated for 1 min. Three independent replicates were analysed at each concentration level.

The LOQ was determined as the lowest concentration at which method precision, expressed as the coefficient of variation (CV), was below 5.0%. LOQ determination was performed using AMX solutions prepared in the blank matrix, consisting of a 50:50 (v/v) mixture of mobile phase and blank formulation extraction solution.

Trueness and precision were evaluated by performing three independent determinations on three different days at two concentration levels corresponding to 80% and 120% of the selected test concentration. Repeatability and intermediate precision were assessed using solutions prepared in the blank matrix. As part of the precision assessment, method robustness and ruggedness were also investigated.

Robustness was evaluated by deliberately varying three chromatographic parameters: mobile phase flow rate, column temperature and UV detection wavelength. The influence of flow rate variations on peak area was assessed by analysing a 0.05 mg/mL AMX solution at flow rates of 0.9, 1.0 and 1.1 mL/min. The effect of minor changes in column temperature was evaluated at 24°C, 25°C and 26°C. Additionally, the impact of small variations in detection wavelength was assessed by comparing peak areas obtained from three independent analyses of the test concentration at 229, 230 and 231 nm.

Method ruggedness was investigated by evaluating analyst‐to‐analyst variability at two AMX concentration levels (0.04 and 0.06 mg/mL). For each concentration level, three analyses were performed by two different analysts over three consecutive days.

Extraction recovery was also evaluated using three gummy tablets prepared with a known amount of penicillin G. These gummy tablets were manufactured and subsequently dispersed, yielding three independent solutions, following the same procedure applied to the AMX‐loaded gummies. Penicillin G was selected as the reference compound due to its chemical and physicochemical similarities to AMX. Quantification of penicillin G in the gummy tablets was performed using a six‐point calibration curve constructed from pure penicillin G standard solutions (in the 0.003–0.1 mg/mL), prepared according to the same protocol used for the AMX standard solutions.

### 2.5 HPLC–UV Analysis of the AMX‐Containing Gummy Tablets

HPLC–UV analyses were performed in triplicate by analysing three independent gummy tablets. Briefly, each gummy tablet was cut into small pieces using a scalpel, dispersed in 1 L of water and stirred at 600 rpm for 24 h. Subsequently, an aliquot of the resulting suspension was centrifuged at 4000 rpm for 10 min at RT. A 0.5 mL aliquot of the supernatant was then diluted with an equal volume of mobile phase. The resulting solution was vortex‐mixed, filtered through a 0.45 µm nylon syringe filter and finally analysed under the chromatographic conditions described in Section 2.2.

### 2.6 Gummy Tablets Stability

To evaluate the stability of AMX in the gummy tablet formulations, long‐term stability studies were conducted. The gummy tablets were stored for a period of 6 months under two different conditions: at 25°C under vacuum in single‐unit packaging and at RT in a dry cabinet. At predefined time points (0, 7, 30, 90 and 180 days), the formulations were cut and dispersed in water as described in Section 2.5, and the AMX content was quantified by HPLC–UV analysis according to the method reported in Section 2.2.

The packaging of medicinal products should be selected to preserve the original physicochemical properties and stability of the drug. Storage at 4°C is commonly adopted for liquid formulations, such as AMX oral suspensions.

In contrast, the formulation investigated in this study was stored under conditions typically applied to solid dosage forms (e.g., tablets), which do not require refrigeration. Solid dosage forms may be packaged in either multidose or unit‐dose formats; however, unit‐dose packaging (e.g., blisters or single‐dose sachets) is generally preferred, as it provides enhanced protection of both the drug and the formulation. Furthermore, to improve stability, air (particularly oxygen, which is responsible for many degradation pathways) is often removed and replaced with inert gases. Under laboratory‐scale conditions, the single‐dose formulation was stored under reduced pressure (vacuum) to mimic, as closely as possible, the storage conditions commonly employed for solid dosage forms.

## 3 Results and Discussion

The initial experimental step consisted of identifying an RP‐HPLC–UV method capable of efficiently analysing the API in the formulation under investigation. To this end, a chromatographic method previously developed by other authors for the determination of AMX in commercial pharmaceutical products and wastewater samples, as well as for the evaluation of its stability in SGF [11], was selected and slightly modified to adapt it to the chromatographic column available in this study. Detailed information on the applied chromatographic conditions is provided in Section 2.2.

Subsequently, the method was validated largely according to ICH Q2(R1) guidelines, following an approach previously reported by some of the present authors [15]. A comprehensive description of the validation parameters and procedures is presented in Section 2.4.

### 3.1 System Suitability

Prior to applying the analytical method to the analysis of real samples, system suitability was assessed by analysing a standard solution of AMX prepared at the test concentration in the mobile phase. System suitability testing was performed to verify that the chromatographic system was operating properly under the selected experimental conditions. All evaluated parameters met the predefined acceptance criteria, confirming the adequacy of the method for routine analysis (see Section 2.2 for further details). Specifically, the tailing factor was 0.95 (acceptance criterion ≤ 2.0), indicating good peak symmetry; the retention factor was 2.42 (acceptance criterion ≥ 2.0), demonstrating appropriate analyte retention; and the number of theoretical plates exceeded 8000 (acceptance criterion ≥ 2000), reflecting satisfactory column efficiency. These acceptance criteria are not defined by ICH guidelines but are frequently specified in United States Pharmacopeia, European Pharmacopoeia and Indian Pharmacopoeia monographs, as well as in validated analytical methods for system suitability assessment [16].

**FIGURE 1:**
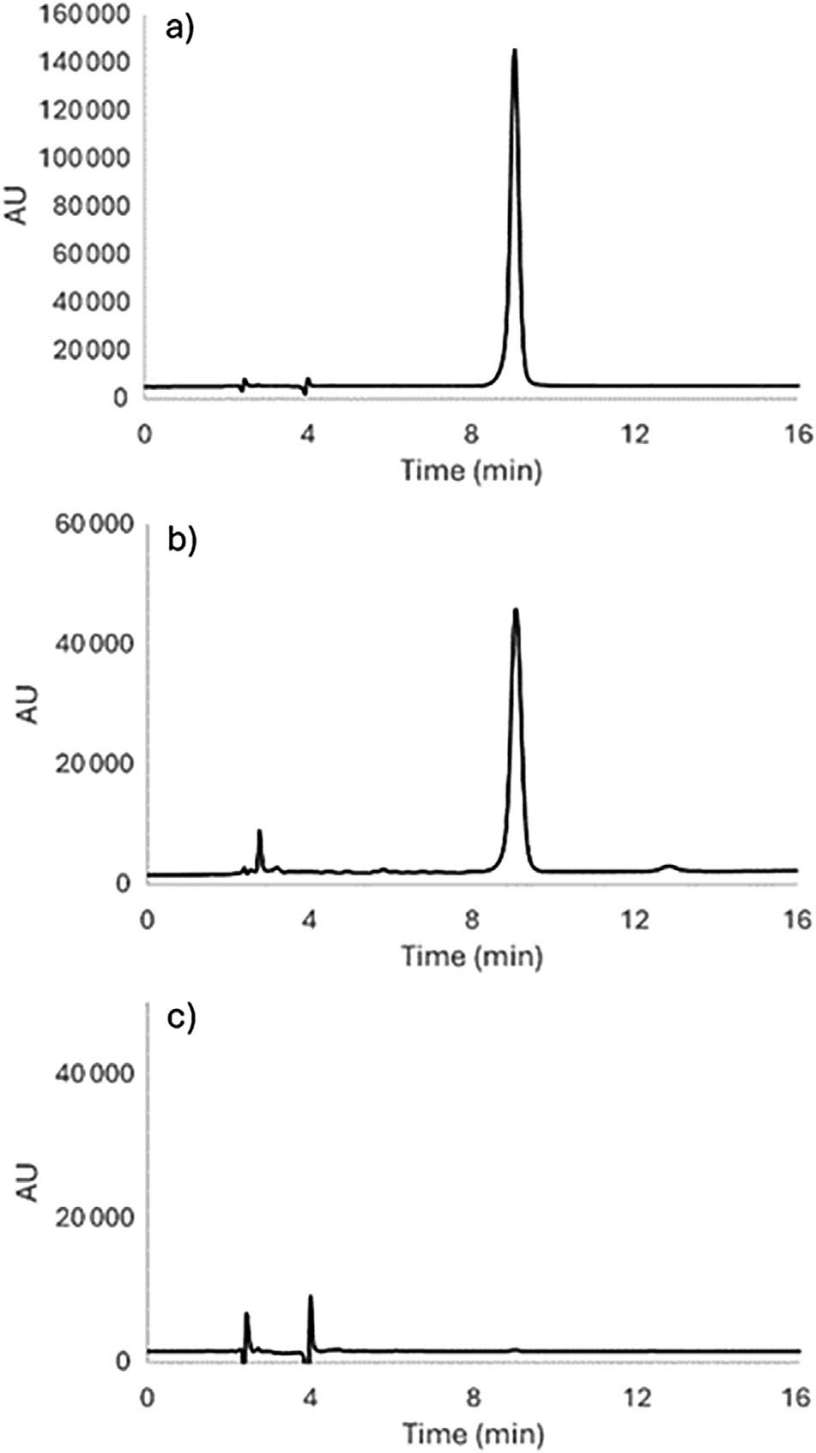
RP‐HPLC chromatograms of (a) AMX trihydrate standard solution, (b) AMX trihydrate extracted from the formulation under investigation, and (c) extraction solution of the blank formulation. Experimental conditions: column, Inertsil ODS‐3 (250 × 4.6 mm^2^ i.d., 5 µm, 100 Å pore size); eluent, 0.1 M K_2_HPO_4_·3H_2_O/ACN (90:10, v/v; pH of the aqueous phase equal to 8.70); flow rate, 1.0 mL/min; column temperature, 25°C; UV at 230 nm. *Y*‐axis is in arbitrary units (AU).

The chromatographic profile obtained from the analysis of an AMX trihydrate standard solution is reported in Figure 1a. For comparison, Figure 1b shows the chromatogram resulting from the analysis of the API extracted from the investigated formulation. The similarity between the two chromatograms further confirms the suitability of the method.

### 3.2 Method Validation

Analysis of standard solutions of AMX trihydrate prepared in the presence and absence of excipients demonstrated that co‐formulants did not influence the chromatographic response. In particular, peak area values obtained from solutions containing the same API concentration showed a similarity greater than 98.5%. Both solutions were prepared at the selected test concentration of AMX using a 50:50 (v/v) mixture of mobile phase and either the blank matrix solution (see Section 2.4 for details) or purified water. Under the applied experimental conditions, the analyte exhibited a retention time of approximately 9 min (Figure 1a,b). Adequate analyte retention is essential to ensure sufficient chromatographic selectivity.

Method specificity was assessed by injecting an extraction solution (i.e., the blank matrix solution) obtained from a blank formulation, defined as a formulation identical to that under investigation but lacking AMX, prepared as described in Section 2.3. No chromatographic peak was observed at the retention time corresponding to AMX trihydrate (Figure 1c).

To define the working range of the developed chromatographic method, a broad concentration interval was evaluated, spanning from 2% (0.0010 mg/mL) to approximately 1000% (0.5 mg/mL) of the selected test concentration. All standard solutions were prepared as described in Section 2.2 for the solution used to construct the calibration curve.

AMX trihydrate standard solutions were prepared by dissolving the API in a 50:50 (v/v) mixture of mobile phase and extraction solution obtained from the blank formulation. For each of the 10 tested concentration levels, the CV% of the peak area values was calculated. On the basis of these results, a narrower concentration range was selected to establish the linearity of the method. This range comprised seven concentration levels within the 0.0039–0.25 mg/mL interval, corresponding to 7.8%–500% of the selected test concentration, and was used to construct the quantitative regression model. This concentration range was chosen to cover approximately two orders of magnitude.

Regression analysis performed using either all 10 concentration levels or the selected seven levels yielded calibration curves with coefficients of determination (*R* ^2^) equal to 0.99. Comparable results were obtained when applying the leave‐one‐out cross‐validation procedure (*R* ^2^_cv). For the seven selected concentration levels, CV% values ≤5% were observed, whereas higher variability was noted at the lowest concentrations (0.0010 and 0.0020 mg/mL) (Table S1). Using the least‐squares approach, the seven‐point calibration curve was described by the following equation:

$$y=21709824\;\left(\pm 83797\right)x+7275\left(\pm 9143\right)$$

where *y* represents the peak area, and *x* represents the analyte concentration. On the basis of the CV% values obtained at the lowest concentrations, the limit of quantification (LOQ) was estimated to lie between 0.0020 and 0.0039 mg/mL. This LOQ was considered satisfactory and appropriate for the intended application of the method.

Method accuracy and precision were evaluated by analysing two concentration levels corresponding to 80% (0.04 mg/mL) and 120% (0.06 mg/mL) of the selected test concentration. The results are reported in Table 1. More details are reported in the Supporting Information document.

**TABLE 1:** Trueness (%), repeatability (%CV_W_), intermediate precision (%CV_IP_) and tolerance interval (%) of the developed method.

Analyte	Theoretical concentration (mg/mL)	Trueness (%)	Repeatability (%CV_W_)	Intermediate precision (%CV_IP_)	Tolerance interval (%)
AMX trihydrate	0.04	102.4	1.60	1.60	100.0–104.7
	0.06	101.3	1.30	1.50	98.95–103.6

*Note*: The tolerance interval here indicated is the statistical range expected to contain a specified proportion of future results with a defined confidence level.

Abbreviation: CV, coefficient of variation.

As reported in Table 1, method trueness, expressed as recovery, was 102.4% at the 0.04 mg/mL concentration level and 101.3% at 0.06 mg/mL. With respect to method precision, the coefficients of variation for repeatability (CV_w_) and intermediate precision (CV_IP_) at both concentration levels were ≤1.6%; detailed results are provided in Tables S2 and S3.

Robustness testing (Table S4) demonstrated that minor variations in detection wavelength did not significantly affect analytical performance, as indicated by a CV% of 1.6%. Similarly, small fluctuations in column temperature had no significant impact on the results, yielding a CV% of 1.1%. In contrast, the flow rate was identified as the only critical parameter, as a ±10% variation resulted in a markedly higher CV% of 8.1%.

Ruggedness evaluation (Table S5) showed that the analytical results were not significantly influenced by analyses performed by two different operators. Peak area values exhibited only minor variations, confirming the ruggedness of the method, with CV% values of 1.7% and 1.4% for the 0.04 and 0.06 mg/mL concentration levels, respectively.

Overall, all validation parameters met the acceptance criteria, indicating that the developed method is reliable and suitable for the quantitative analysis of AMX in the investigated innovative formulation.

The recovery of the API was initially assessed by determining the AMX content in the gummy tablets and comparing it with the corresponding theoretical value. Within the context of these experiments, a recovery consistently exceeding 94% was observed. To further confirm the quantitative recovery of the active compound from the pharmaceutical formulation, the same experimental approach was subsequently applied using penicillin G.

Gummy tablets containing penicillin G were analysed using the RP‐HPLC–UV method described in Section 2.2 in order to evaluate the extraction recovery. Penicillin G was selected as a model compound due to its chemical and physicochemical similarities to AMX. Methodological details regarding the preparation of penicillin G‐containing gummy tablets and the concentrations used for the standard solutions are reported in Sections 2.3 and 2.4, respectively. Analysis of the penicillin G gummy tablets yielded an extraction recovery of 97.0% ± 0.1%.

### 3.3 Analysis of the AMX‐Containing Gummy Tablets and Stability Evaluation

The validated method was finally applied to analyse the AMX‐containing gummy tablets, prepared as described in Section 2.5. The total content of AMX in tablets was found to be 27.9 ± 1.1 mg, corresponding to 94.7% ± 3.7% of the expected amount.

The stability profile of AMX in the gummy tablet matrix (Table 2) indicates a progressive loss of chromatographically detectable active substance over the 180‐day monitoring period under both storage conditions tested. At the initial 7‐day time point, AMX content expressed as a percentage of the initial peak area declined more steeply in samples stored under vacuum at 25°C (81.9%) compared with those maintained in a dry cabinet at RT (94.5%), suggesting that the vacuum environment and associated microenvironmental factors may enhance degradation pathways early in the storage period. This trend of greater degradation under vacuum persisted through the longest storage interval, with residual AMX levels of 58.6% under vacuum versus 70.3% in dry cabinet storage at 180 days (Table 2).

**TABLE 2:** Stability of gummy tablets as a function of time under different storage conditions (dry cabinet at room temperature and under vacuum at 25°C), expressed as % of initial area.

Time (days)	% of initial area	
	Dry cabinet at RT	Under vacuum at 25°C
0	100.0	100.0
7	94.5	81.9
30	92.9	76.2
90	72.1	75.4
180	70.3	58.6

Abbreviation: RT, room temperature.

These observations are consistent with the well‐documented chemical lability of AMX under non‐refrigerated storage conditions and the influence of environmental factors on degradation kinetics. AMX is known to undergo hydrolytic degradation via opening of the β‐lactam ring, with rates that increase with temperature and in the absence of stabilizing conditions [17]. Similarly, longer term stability studies on reconstituted AMX suspensions at various storage temperatures demonstrate that degradation proceeds more rapidly at higher temperatures, with concentrations falling below 80% of initial values within 1–2 weeks when refrigeration is not maintained. These findings support the trend observed in the gummy tablets, where elevated storage temperatures and potentially altered moisture interaction under vacuum could accelerate degradation pathways relative to ambient dry cabinet conditions.

Importantly, the retention of a higher fraction of AMX in the dry cabinet at RT compared to vacuum storage suggests that factors, such as moisture diffusion, residual water activity in the gummy matrix and potential shifts in microenvironment pH, may play a role in stabilizing the β‐lactam ring in the absence of extreme desiccation. The gummy tablet formulation inherently includes hygroscopic excipients that could moderate local water activity and thus slow hydrolytic degradation relative to fully desiccated or highly stressed storage conditions. Although direct comparisons to solid oral dosage forms such as conventional tablets are limited, accelerated and real‐time stability studies of AMX solid dosage forms traditionally aim to demonstrate maintenance of assay within pharmacopoeia limits (90%–110%) over long‐term storage at controlled temperature and humidity.

## 4 Conclusions

In this study, a robust and reliable RP‐HPLC–UV method was successfully applied and validated for the quantitative determination of AMX in an innovative gummy tablet formulation intended for paediatric use. The method was validated in accordance with ICH Q2(R1) guidelines, demonstrating adequate specificity, linearity, sensitivity, trueness, precision, robustness and ruggedness. The use of a blank matrix derived from the blank formulation was useful in realistically mimicking the sample matrix and ensuring the reliability of quantitative analyses. In addition, extraction recovery was satisfactorily assessed using penicillin G as a model compound, yielding a recovery of approximately 97%, thus confirming the suitability of the extraction procedure.

Application of the validated method to the analysis of AMX‐loaded gummy tablets confirmed that the manufacturing process resulted in a drug content close to the theoretical value, supporting the feasibility of this formulation approach. Stability studies further revealed that AMX undergoes progressive degradation within the gummy matrix over time with storage conditions playing a significant role. In particular, storage under vacuum at 25°C was found to be less favourable, leading to a more pronounced decline in AMX content compared with storage in a dry cabinet at RT.

Overall, the results demonstrate that the developed RP‐HPLC–UV method is suitable for routine quality control and stability assessment of AMX‐containing gummy tablets. Moreover, the findings highlight the potential of gummy tablets as a ‘child‐friendly’ dosage form, while also emphasizing the need for further formulation and packaging optimization to enhance long‐term stability. This work may contribute to the advancement of analytical strategies and formulation development for innovative paediatric antibiotic therapies.

## Author Contributions

**Samuele Bonafè**: methodology, investigation, writing – original draft preparation. **Anna Imbriano**: methodology, investigation, writing – original draft preparation. **Anna Migni**: investigation. **Elisa Bianconi**: investigation. **Laura Mercolini**: writing – review and editing. **Luana Perioli**: writing – review and editing, found acquisition. **Cinzia Pagano**: writing – review and editing, found acquisition. **Roccaldo Sardella**: conceptualization, methodology, validation, formal analysis, writing – original draft preparation, supervision, found acquisition.

## Conflicts of Interest

The authors declare no conflicts of interest.

## Supporting information




**Supporting File**: elps70103‐sup‐0001‐SuppMat.docx.

## Data Availability

The data that support the findings of this study are available from the corresponding author upon reasonable request.
