# Supplementary material for: Validated RP‐HPLC–UV Method for the Quantitative Analysis and Stability Evaluation of Amoxicillin in Paediatric Gummy Tablets
Source: Electrophoresis. 2026 May 3;47(7):605–12. doi: 10.1002/elps.70103 (PMC13378233; doi:10.1002/elps.70103)
Supplement: Supplementary file 1 — Supporting File: elps70103‐sup‐0001‐SuppMat.docx. [file ELPS-47--s001.docx]

**SUPPORTING INFORMATION**

**Validated RP-HPLC–UV Method for the Quantitative Analysis and Stability Evaluation of Amoxicillin in Paediatric Gummy Tablets**

*Samuele Bonafè^1a^, Anna Imbriano^1a^, Cinzia Pagano^1^, Anna Migni^1^, Elisa Bianconi^1^,*

*Laura Mercolini^2^, Luana Perioli^1^, Roccaldo Sardella^1^**

^a^ Samuele Bonafè and Anna Imbriano contributed equally to the work

^1^Department of Pharmaceutical Sciences, University of Perugia, Via del Giochetto, 06122 Perugia, Italy.

^2^Department of Pharmacy and Biotechnology (FaBiT), Alma Mater Studiorum, University of Bologna, Via Belmeloro 6, 40126 Bologna.

*Corresponding author:

Roccaldo Sardella, email: roccaldo.sardella@unipg.it

**Table S1: Regression data obtained at all ten considered concentration levels. Three consecutive analyses have been executed at each of the selected concentration values. Peak area values are reported in Arbitrary Units (AU). Experimental conditions are reported in detail in Section 2.2 of the Main Document.**

| Conc. (mg/mL) | **Peak Area 1 (AU)** | **Peak Area 2 (AU)** | **Peak Area 3 (AU)** | **Average Peak Area (AU)** | St. Dev. | CV% |
| --- | --- | --- | --- | --- | --- | --- |
| 0.5000 | 10786677 | 10749710 | 10937669 | 10824685 | 81304 | 0.8 |
| 0.2500 | 5513620 | 5452552 | 5332266 | 5432813 | 75342 | 1.4 |
| 0.1250 | 2749905 | 2725834 | 2695653 | 2723797 | 22195 | 0.8 |
| 0.0625 | 1372091 | 1368106 | 1353137 | 1364445 | 8160 | 0.6 |
| 0.0313 | 701528 | 687320 | 681511 | 690120 | 8408 | 1.2 |
| 0.0156 | 353446 | 346494 | 340002 | 346647 | 5490 | 1.6 |
| 0.0078 | 181801 | 174169 | 169403 | 175124 | 5106 | 2.9 |
| 0.0039 | 93141 | 86971 | 84140 | 88084 | 3758 | 4.3 |
| 0.0020 | 50490 | 43131 | 40962 | 44861 | 4078 | 9.1 |
| 0.0010 | 25984 | 21915 | 20041 | 22647 | 2481 | 11.0 |

**Table S2: Validation data: recovery values (%) calculated at two concentration levels for three consecutive days as (C_measured_/C_theoretical_)*100. Three consecutive analyses have been executed at each of the selected concentration values. Experimental conditions are reported in detail in Section 2.2 of the Main Document. Peak area values are reported in Arbitrary Units (AU).**

| **Theoretical** Concentration (mg/mL) | **Peak Area (AU)** | Calculated Concentration (mg/mL) | **Average Calculated Concentration (mg/mL)** | Std. Dev. | Recovery % |
| --- | --- | --- | --- | --- | --- |
| 0.04 (day 1) | 886893 | 0.0405 | 0.0407 | 0.0005 | 101.7 |
|  | 905204 | 0.0414 |  |  |  |
|  | 877993 | 0.0401 |  |  |  |
| 0.06 (day 1) | 1344271 | 0.0616 | 0.0614 | 0.0001 | 102.3 |
|  | 1336816 | 0.0612 |  |  |  |
|  | 1338913 | 0.0613 |  |  |  |
| 0.04 (day 2) | 892298 | 0.0408 | 0.0412 | 0.0005 | 102.9 |
|  | 894630 | 0.0409 |  |  |  |
|  | 915369 | 0.0418 |  |  |  |
| 0.06 (day 2) | 1340702 | 0.0614 | 0.0608 | 0.0008 | 101.4 |
|  | 1304335 | 0.0597 |  |  |  |
|  | 1338914 | 0.0613 |  |  |  |
| 0.04 (day 3) | 881298 | 0.0403 | 0.0410 | 0.0006 | 102.6 |
|  | 902150 | 0.0412 |  |  |  |
|  | 910677 | 0.0416 |  |  |  |
| 0.06 (day 3) | 1321820 | 0.0606 | 0.0601 | 0.0008 | 100.2 |
|  | 1327261 | 0.0608 |  |  |  |
|  | 1287982 | 0.0590 |  |  |  |

**Table S3: Validation data: intermediate precision calculated at two concentration levels in the time-frame of three consecutive days.**

| Concentration (mg/mL) | Average of three consecutive days (mg/mL) | Std. Dev. |
| --- | --- | --- |
| 0.04 | 0.0409 | 0.00021 |
| 0.06 | 0.0608 | 0.000521 |

**Table S4: Validation data: robustness evaluated considering a subtle variation of the three experimental variables, flow rate (1.0 ± 0.1 mL/min), wavelength of detection (230 ± 1 nm), and column temperature (25 ± 1 °C). Three consecutive analyses have been executed at each of the selected concentration values. Area values are reported in Arbitrary Units (AU). Experimental conditions are reported in detail in Section 2.2 of the Main Document.**

| Flow rate (mL/min) | **Peak Area (AU)** |
| --- | --- |
| 0.9 | 1253982 |
|  | 1242822 |
|  | 1218375 |
| 1 | 1171068 |
|  | 1098441 |
|  | 1092038 |
| 1.1 | 1009963 |
|  | 1020745 |
|  | 1032032 |
| Std. Dev. | 91817.34 |
| Avg. Area | 1126607 |
| CV% | 8.1 |

| Wavelenght (nm) | **Peak Area (AU)** |
| --- | --- |
| 229 | 1122017 |
|  | 1120605 |
|  | 1132154 |
| 230 | 1094213 |
|  | 1092220 |
|  | 1104606 |
| 231 | 1081582 |
|  | 1079376 |
|  | 1091644 |
| Std. Dev. | 17811.2 |
| Average Area | 1102046 |
| CV% | 1.6 |

| Temperature (°C) | **Peak Area (AU)** |
| --- | --- |
| 24 | 1085870 |
|  | 1069114 |
|  | 1087515 |
| 25 | 1094213 |
|  | 1092220 |
|  | 1104606 |
| 26 | 1109674 |
|  | 1099183 |
|  | 1103993 |
| Std. Dev. | 11621.57 |
| Average Area | 1094043 |
| CV% | 1.1 |

**Table S5: Validation data: ruggedness evaluated considering the analytical results obtained at two concentration levels by two different analysts over three consecutive days. Area values are reported in Arbitrary Units (AU). Experimental conditions are reported in detail in Section 2.2 of the Main Document.**

| Conc. (mg/mL) | **Peak Area (AU)** | **Average Peak Area (AU)** | CV% |
| --- | --- | --- | --- |
| 0.04 | 860571 | 838035.2222 | 1.7 |
|  | 836067 |  |  |
|  | 829125 |  |  |
|  | 828949 |  |  |
|  | 825118 |  |  |
|  | 816002 |  |  |
|  | 844007 |  |  |
|  | 835863 |  |  |
|  | 841749 |  |  |
|  | 841212 |  |  |
|  | 841306 |  |  |
|  | 842409 |  |  |
|  | 864363 |  |  |
|  | 825126 |  |  |
|  | 856105 |  |  |
|  | 827987 |  |  |
|  | 856982 |  |  |
|  | 811693 |  |  |
| 0.06 | 1300542 | 1293149 | 1.4 |
|  | 1315477 |  |  |
|  | 1319090 |  |  |
|  | 1269924 |  |  |
|  | 1270941 |  |  |
|  | 1311313 |  |  |
|  | 1282684 |  |  |
|  | 1307240 |  |  |
|  | 1296286 |  |  |
|  | 1315579 |  |  |
|  | 1303281 |  |  |
|  | 1307660 |  |  |
|  | 1255827 |  |  |
|  | 1290261 |  |  |
|  | 1295618 |  |  |
|  | 1273877 |  |  |
|  | 1289981 |  |  |
|  | 1271101 |  |  |
